# Combining transcriptomics and genetic linkage based information to identify candidate genes associated with *Heterobasidion*-resistance in Norway spruce

**DOI:** 10.1038/s41598-020-69386-0

**Published:** 2020-07-29

**Authors:** Rajiv Chaudhary, Karl Lundén, Kerstin Dalman, Mukesh Dubey, Miguel Nemesio-Gorriz, Bo Karlsson, Jan Stenlid, Malin Elfstrand

**Affiliations:** 1 Department of Forest Mycology and Plant Pathology, Swedish University of Agricultural Sciences, Box 7026, 75007 Uppsala, Sweden; 2 Department of Molecular Sciences, Swedish University of Agricultural Sciences, 75007 Uppsala, Sweden; 3 Teagasc Food Research Centre, Ashtown, Dublin 15, D15DY05 Ireland; 4 The Forestry Research Institute of Sweden, 268 90 Ekebo, Svalöv, Sweden

**Keywords:** Genetics, Plant sciences

## Abstract

The *Heterobasidion annosum* s.l species complex comprises the most damaging forest pathogens to Norway spruce. We revisited previously identified Quantitative Trait Loci (QTLs) related to *Heterobasidion*-resistance in Norway spruce to identify candidate genes associated with these QTLs*.* We identified 329 candidate genes associated with the resistance QTLs using a gene-based composite map for *Pinaceae.* To evaluate the transcriptional responses of these candidate genes to *H. parviporum,* we inoculated Norway spruce plants and sequenced the transcriptome of the interaction at 3 and 7 days post inoculation. Out of 298 expressed candidate genes 124 were differentially expressed between inoculation and wounding control treatment. Interestingly, *PaNAC04* and two of its paralogs in the subgroup III-3 of the NAC family transcription factors were found to be associated with one of the QTLs and was also highly induced in response to *H. parviporum.* These genes are possibly involved in the regulation of biosynthesis of flavonoid compounds. Furthermore, several of the differentially expressed candidate genes were associated with the phenylpropanoid pathway including a *phenylalanine ammonia-lyase*, a *cinnamoyl-CoA reductase*, a *caffeoyl-CoA O-methyltransferase* and a *PgMYB11*-like transcription factor gene. Combining transcriptome and genetic linkage analyses can help identifying candidate genes for functional studies and molecular breeding in non-model species.

## Introduction

One of the most pressing challenges to the forest industry is facing the increasing damages caused by forest pests and pathogens^1,2^. Although good management practices can reduce the problems^3,4^, examining and utilizing the genetic resistance, through forest tree breeding, offers an additional powerful method to reduce damages^1,3^.

Norway spruce [*Picea abies* (L.) Karst.] is an important conifer species for the forest industry in Europe^2^. For instance, it constitutes 41% of the standing Swedish tree volume^5,6^. The major pathogen, in economic terms^2^, on Norway spruce is the species complex *Heterobasidion annosum *sensu lato (s.l.), which causes stem and root-rot in Europe^7,8^. Although new airborne infections of *H. annosum* s.l. can be reduced by good forest management and stump treatment with the biocontrol agent *Phlebiopsis gigantea*^9^, carryover between rotations in already infected sites is an important factor for the health of the new generation trees^10^. Thus, replantation of plant material with improved resistance would be a valuable resource in the management of *H. annosum* s.l. in Norway spruce.

Genetic variation for resistance to *H. annosum* s.l. exists in Norway spruce^11–16^ and there are no adverse correlations between resistance to *Heterobasidion* infection and growth or wood quality traits^12,13,16^. Hence, selection for resistance to *H. annosum* s.l. in breeding programs could lead to considerable gain^12^, without compromising other breeding achievements. Resistance to wood rotting pathogens, such as *H. annosum* s.l. in conifers, presents a challenge to tree breeders as the disease often manifests late in the tree’s life, which makes phenotypic selection difficult and time consuming. Marker Assisted Selection (MAS) holds promises for increasing the gain in tree breeding and specifically in resistance breeding^17^. Yet, MAS has not been widely implemented within tree breeding programs mainly due to the difficulty in translating Quantitative Trait Loci (QTLs) analysis into operational MAS i.e. validation of potential markers^17,18^. Recently, one of the marker candidates, *PaLAR3* was identified in the first Quantitative Trait Locus (QTL) analysis for resistance against *H. parviporum* (Fr.) Niemelä & Korhonen^14,15^*,* which is a member of the *H. annosum* s.l. complex and lives almost exclusively in Norway spruce^19^. The validation of *PaLAR3*, a gene that encodes for a leucoanthocyanidin reductase that is involved in the flavonoid-biosynthetic pathway^20^, was done through the integration of information of phenotypic, transcriptional, metabolic, and genetic evidence^15^. Similarly, two other reported markers that are ready to be used for MAS of trees with improved resistance to pathogens and pests in conifers^21,22^; build on the integration of genetic information and complementing techniques.

The QTL analysis in Norway spruce for resistance to *H. parviporum* identified 13 QTLs linked with four traits related to host resistance^14^: *lesion length* at the inoculation site (LL), *exclusion* of the pathogen from the host after initial infection (E), *infection prevention* from establishing at all (IP) and *fungal spread within the sapwood* (SWG). The validated marker, *PaLAR3,* comprised one of the four QTLs associated with SWG^14,15^. In this study, we aimed to use the high degree of synteny and macrocollinearity within *Pinaceae* to identify novel candidate resistance genes associated with the QTLs for resistance to *H. parviporum*^14^. We used the composite map of the *Pinaceae* family and gene expression patterns in Norway spruce after challenging it with *H. parviporum*; therefore, we could identify candidate resistance genes for future validation and functional analyses.

First, we used a *Pinaceae* composite linkage map to identify additional Norway spruce candidate genes associated with already described resistance QTLs. Second, we predicted that the combination of genetic and transcriptional information, would enable us to identify candidate resistance genes of induced response for future analyses; allowing us to hypothesize that the genes in the QTL regions, which are likely to be important for controlling spread of *H. parviporum,* are also likely to be more strongly regulated by inoculation than by wounding alone. Previous studies have highlighted broad similarities in defence responses to *H. parviporum* infection and to wounding control compared to naïve material, although inoculated samples showed a heightened response^23,24^.

The composite map of the *Pinaceae* family^25^ integrated published maps of Norway spruce^14^, *Picea glauca* and *Picea mariana*^26^ and *Pinus taeda*^27^ with genetic maps from multiple crosses of *Pinus pinaster*. QTL markers from the Norway spruce linkage map^14^ were included in the *Pinaceae* composite map^25^. As the composite map is considerably denser than the Norway spruce map we chose to focus on markers in and around a significant QTLs for resistance identified by Lind et al.^14^ to identify candidate genes in the *Pinaceae* composite map^25^. This summed up to 329 candidate genes associated with 12 genomic regions of Norway spruce. We further determined the transcriptional responses of these candidate genes at three and seven days after inoculation with *H. parviporum.* We detected 124 differentially expressed candidate resistance genes, including two putative NAC family transcription factor genes in response to *H. parviporum* inoculation compared to the control treatment of mechanical wounding.

## Materials and methods

### Identification of conifer candidate genes associated with QTL regions from a composite map

The markers in the Norway spruce QTL map^14^ were integrated in the *Pinaceae* composite map^25^. The markers in the Norway spruce QTL map^14^ were used to identify the corresponding QTL regions in the *Pinaceae* composite map^25^. The markers associated with the QTL regions were identified around a significant QTL LOD-peak which contained significant markers (*P* value < 0.05) according to the Kruskal–Wallis test in Norway spruce linkage map^14^. The candidate genes between significant markers in the confidence interval, in the *Pinaceae* composite map were identified and selected as confidence interval candidate genes (CCGs) for future analyses (Supplementary file1). The candidate genes in between the next subsequent markers outside the confidence interval were also selected. These genes were referred as putative candidate genes (PCGs) (Supplementary file1). We chose to include PCGs because all the markers used in the Norway spruce QTL map^14^ were not included in the *Pinaceae* composite map^25^. For some QTL regions this discripancy made it difficult to delineate the QTL region, thus the genes in the category represent genes which are suspected to associate with the QTL region. Therefore, differentially expressed PCGs could still be interesting in this experimental set up as a part of an induced defence system.

### Identification of Norway spruce candidate resistance genes associated with QTL regions

The FASTA sequences of the unigenes derived markers corresponding to genes in regions of the genome associated with the resistance QTLs were downloaded from the *P. pinaster* unigene catalogue (https://www.scbi.uma.es/sustainpinedb/unigens) and the most probable Norway spruce orthologues were identified by a blastN query (E-value cutoff:1e-3) in the Norway spruce gene catalogue (*Pabies* v1.0, www.congenie.org), excluding low confidence candidate genes with less than 30% coverage.

### Norway spruce materials

For the RNAseq study, six 7-year-old rooted cuttings of each of the genotypes S21K0220126 and S21K0220184, originating from a well-studied full-sib family (S21H9820005) of Norway spruce^11,14,23^, were used. The cuttings were grown in a greenhouse with an 18 h light regime. Watering and nutrients were supplied twice a week. The Real-Time Quantitative Reverse Transcription PCR (qRT-PCR) validation experiment included cuttings of six genotypes (S21K0220263, S21K0220240, S21K0220237, S21K0220161, S21K022136 and S21K022346) from the same Norway spruce full-sib family^23^.

### Inoculation experiment

Branches were artificially inoculated with *H. parviporum* (isolate Rb175) as previously described^23^. The same isolate was used to generate the QTL map in Norway spruce^14^. Briefly, branches were wounded with a five-mm cork borer and wooden plugs covered with mycelium from *H. parviporum* were attached to the wound with Parafilm®; control branches on the tree were also wounded and a sterile wooden plug was attached and sealed with Parafilm.

Based on the difference in necrotic lesion length (LL) extension from the inoculation site after inoculation with *H. parviporum* the genotypes S21K0220126 and S21K0220184 (short and long, respectively, data not shown) were selected for RNA sequencing. For the RNAseq study, bark and phloem samples were harvested at three and seven days post-inoculation (dpi). At the time of harvest, bark surrounding the wounds and inoculation sites was cut into two sections and samples were collected at the inoculation site (A) 0–0.5 cm around the wound, and distal to the inoculation site (C) 1.0–1.5 cm. We used six ramets per clone and three inoculations per twig were done. The bark samples were frozen separately in liquid nitrogen and stored at − 80˚C until further use. For the qRT-PCR study sampling is described in detail elsewhere^23^, briefly one bark and phloem sample was taken for each treatment and time point from six separate full-sib genotypes.

### RNA extraction, transcriptome sequencing and qRT-PCR

#### RNA extraction

Total RNA was isolated by using a modified CTAB extraction protocol^28^. The samples were treated with DNase I (Sigma-Aldrich) to eliminate contamination of genomic DNA. The RNA integrity was analysed by using the Agilent RNA 6,000 Nano kit (Agilent Technologies Inc.).

### Transcriptome sequencing and bioinformatics analyses

Three biological replicates of clones S21K0220126 and S21K0220184 per treatment were used for Illumina sequencing. Sequencing libraries were prepared at the SNP&SEQ Technology Platform (SciLifeLab, Uppsala) using the TruSeq stranded mRNA sample preparation kit according to the manual TruSeq stranded mRNA sample preparation guide. Sequencing was done using HiSeq 2,500, paired-end 125 bp read length, v4 sequencing chemistry. The raw sequences were submitted to the Sequence Read Archive (SRA) portal (NCBI) under BioProject accession number PRJNA522265.

RNAseq analyses were performed with the *Tophat-Cufflinks* pipeline as previously described^29^. Briefly, *Nesoni* clip 0.97 (https://github.com/Victorian-Bioinformatics-Consortium/nesoni) was used to filter adaptors and low-quality bases. Illumina reads were filtered based on phred-scale with a quality score cut-off of 20, minimum adapter length match of 20, with maximum errors of one in the adaptor and reads shorter than 35 were discarded. A *Bowtie* reference from the ‘*Pabies*1.0-all-cds.fna’ was constructed, downloaded from the Norway spruce genome portal (https://congenie.org/) using *Bowtie2* version 2.2.4 (https://bowtie-bio.sourceforge.net/bowtie2/index.shtml) to enable alignments to a reference database. The filtered read pairs were aligned to ‘*Pabies*1.0-all-cds’ reference gene model with *Tophat* version 2.0.13^30^. *Cufflinks* version 2.2.1 was used to assemble all transcripts of each sample with the results of the alignment from *TopHat. Cuffmerge* included in the *cufflinks* package was used to merge all assemblies. *Cuffquant* (https://coletrapnell-lab.github.io/cufflinks/manual/) calculated transcript abundance from the single assembly of the sample, and the aligned read files produced by the *Tophat* output were run separately for each sample. *Cuffdiff* was used for differential expression analysis using default settings^30,31^.

### qRT-PCR

One µg of total RNA was reverse transcribed to cDNA with the iScript cDNA Synthesis Kit (Bio-Rad) in a total reaction volume of 20 μl according to the manufacturer’s instructions. A ten-fold dilution of the cDNA was stored at − 20 °C. cDNA equivalent to 25 ng of total RNA worked as template for each PCR reaction, using SSoFast EVAGreen Supermix (Bio-Rad). Primers for candidate genes were designed within the exons of the predicted candidate genes in the *P. abies* v 1.0 release using Primer3 software^32^ with a melting temperature (Tm) between 60 °C and 61 °C. A final concentration of 0.15 μM of each primer (Supplementary Table 1) was used. The thermal-cycling condition parameters, ran on an iQ™5 Multicolor Real-Time PCR Detection system (Bio-rad) using the following cycling parameters: 98 °C for 2 min; 40 cycles of 98 °C for 5 s, 60 °C for 10 s. A melt-curve analysis followed the qRT-PCR reactions, to confirm that the signal was the result of a single product amplification. Primer amplification efficiency was determined by amplification of serial dilutions of cDNA from Norway spruce with PCR conditions described above. The relative expression was calculated from threshold cycle values (Ct) using the 2ΔΔCT-method^33^. Transcript abundance was normalized to the reference genes *eukaryotic translation initiation factor 4A* (*elF4A*)^34^ and *elongation factor 1-α* (*ELF1α*)^23^. The gene expression experiments were done with six biological and two technical replicates. Gene expression data were analysed by analysis of variance (ANOVA) using a general linear model approach implemented in R-program (https://www.r-project.org/).

## Results

### Markers and candidate genes associated with resistance QTLs

To identify additional Norway spruce unigene derived SNPs markers in the already described resistance QTLs, we identified 369 *P. pinaster* unigene derived SNPs markers in the composite map^25^ based on markers in the QTLs in Norway spruce^14^ in the composite map^25^ (Table 1). The *P. pinaster* unigene derived SNPs markers were used to query the Norway spruce gene catalogue. A total of 329 candidate genes previously not known to associate with the resistance QTLs were successfully identified, of which 83 were CCGs in between the significant markers within the confidence interval and 246 were PCGs in between the subsequent markers outside the confidence interval in *Pinaceae* composite map^25^ (Table 1). Twelve out of the original 13 QTLs were identified, and only the QTL for LL on linkage group (LG) 9 could not be identified in the composite map (Table 1).

**Table 1:** Markers and candidate genes distributed among Norway spruce linkage groups.

LG^a^	QTL^b^	Markers^c^			Candidate gene^d^		
		CCG	PCG	Total	CCG	PCG	Total
LG1	IP	12	0	12	12	0	12
	E	3	18	21	3	15	18
LG2	IP	15	3	18	13	3	16
	E	3	48	51	3	41	44
	SWG	1	13	14	1	12	13
LG3	E	1	18	19	1	17	18
LG6	SWG1	1	29	30	1	25	26
	E	23	97	120	20	84	104
	SWG2	14	3	17	14	3	17
LG8	LL	1	12	13	1	12	13
LG9	SWG	9	8	17	8	8	16
	LL	0	0	0	0	0	0
LG11	IP1	5	31	36	5	26	31
	IP2	1	0	1	1	0	1
Total		89	280	369	83	246	329

^a^Linkage groups are numbered according to the Lind et al.^14^.

^b^QTL regions for traits controlling resistance to *H. parviporum* as described in Lind et al.^14^: exclusion (E), infection prevention (IP), lesion length (LL), sapwood growth of fungus (SWG).

^c^The number of unique SNP markers in the QTL regions reported by de Miguel et al.^25^.

^d^The number of unique candidate genes in Norway spruce genome these markers correspond to. “CCG” and “PCG” stands for candidate genes within the confidence interval and outside the confidence interval respectively.

### Transcriptome analysis

**Figure 1:**
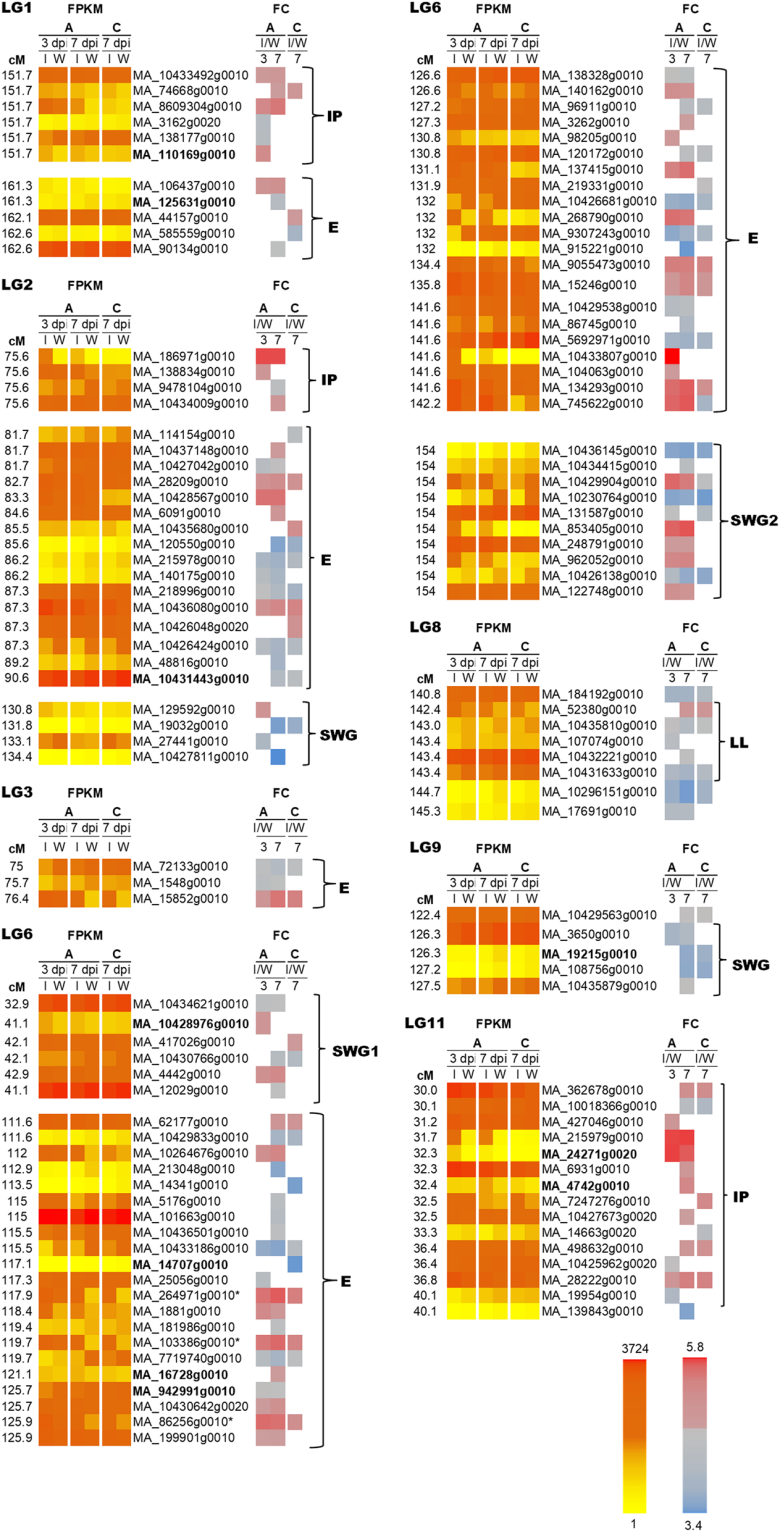
Heat map of the 124 differentially expressed candidate genes associated with *Heterobasidion*-resistance QTLs in Norway spruce in response to *H. parviporum.* The red to yellow colours indicate the highest to lowest FPKM (fragments per kilo base of exon model per million reads mapped) values at 3 and 7 dpi at the proximal (**A**) and distal (**C**) sampling sites. The red-grey-blue scale shows the highest to lowest Fold change (FC, log_2_ values) in *H. parviporum* inoculation compared to wounding alone. Bold font indicate candidate genes corresponding to the original QTL on the Linkage groups, “E” stands for exclusion, “IP” stands for infection prevention, “LL” stands for lesion length and “SWG” stands for fungal growth in sapwood. Asterisks (*) indicates subgroup III-3 NAC TFs. cM indicates the position in centiMorgans of the marker in the *Pinaceae* composite map.

The Illumina HiSeq sequencing generated 14.2–17.8 M reads per sample that passed Illumina’s quality control and between 12.6 M and 16.4 M read-pairs were kept after *Nesoni* filtering (Supplementary file 2). The read mapping rate from *Tophat* was 44.3%–34.9% (Supplementary file 3). Depending on the contrast, between 4,401 and 8,767 genes were significantly differentially expressed (Supplementary Table 2). The fractions of induced and repressed genes were similar in all comparisons. Of the 329 candidate genes selected in the *Pinaceae* composite map^25^, 298 were expressed (80 in CCG and 218 in PCG categories respectively), in at least one of the treatments in the RNAseq experiment (Supplementary Table 3). The candidate genes showed differentially expression at three and seven dpi after *H. parviporum* inoculation compared to wounding, of which 41 were differentially expression in CCG and 83 in PCG categories in the treatments in RNAseq experiment (Fig. 1, Supplementary Table 3).

### DEGs in QTLs associated with infection prevention

The QTLs associated with IP positioned on LG1, LG2 and LG11^14^ were identified in the composite map. Twelve expressed and six differentially expressed candidate genes (DEGs) associated with the QTL on LG1 (Fig. 1, Supplementary file 4). Four were upregulated adjacent to the inoculation site and two DEGs were downregulated at three dpi proximal to the inoculation site compared to wounding (Fig. 1). Most expression changes were quite moderate at LG1 with maximum log_2_ fold change of 1.75.

Fifteen expressed genes were associated with IP on LG2 (Supplementary file 4). Twelve of these were classified as CCGs and three as PCGs. None of PCGs were differentially expressed (Supplementary file 4). Four DEGs were associated with IP on LG2 were identified as CCGs (Fig. 1, Supplementary file 4). Two DEGs were upregulated at three dpi at the inoculation site, and one DEG was still upregulated at seven dpi. Again, most changes in expression patterns were quite moderate on LG2, except a putative *glycosyltransferase* gene (MA_186971g0010), an ortholog of the *Arabidopsis* protein UGT85A1 that encodes a UDP-Glycosyltransferase (UGT) protein. This gene has an interesting expression pattern, it is highly upregulated around the inoculation site at three and seven dpi, although its expression level was low (Fig. 1; Supplementary file 4). It should be mentioned that a second *UGT* like gene, MA_10436196g0010 was among the identified candidate genes. MA_10436196g0010 correspond to the original marker 0.276-A13-934 significantly associated with SWG on LG9^14^, but this candidate gene was not differentially expressed (Table 2, Supplementary file 4).

Linkage group 11 associated with a QTL for IP contained a total of 29 expressed genes and 15 DEGs (Fig. 1; Supplementary file 4). Only five of the expressed genes were categorized as CCGs, three of these were differentially expressed. Interestingly, the *PgMYB11*-like (*R2R3-MYB transcription factor PgMYB11-like*) candidate gene MA_24271g0020/BT103501, associating with the QTL in the original study^14^, was highly upregulated at both three and seven dpi adjacent to the pathogen inoculation site (Fig. 1, Supplementary file 4). Likewise, the candidate gene MA_4742g0010 (PabiesFT1-1,251/BT115191 encoding spruce *Mother of FT1, MFT1*), was also positioned in this QTL in the original study. It was up-regulated in response to the inoculation at seven dpi even though its expression level was generally low (Fig. 1, Supplementary file 4). The candidate gene MA_6931g0010 encoding a putative *caffeoyl-CoA O-methyltransferase* (*CCoAOMT)* gene was highly expressed in all treatments and was differentially upregulated at seven dpi proximal to the inoculation site. It is noteworthy, that all three DEGs *PgMYB11*-like, *Mother of FT1, MFT1, caffeoyl-CoA O-methyltransferase* were identified as CCGs.

### DEGs in QTLs associated with fungal sapwood growth

The QTLs for SWG were located on LG2, LG6 and LG9 in the original study^14^. Twenty five candidate genes associated with SWG QTLs were differentially expressed in our experiment. Four DEGs, associated with the marker BT105733, showed differential regulation found on LG2. Three DEGs were clearly downregulated adjacent to the inoculation site (Fig. 1; Supplementary file 4).

LG6 has two separate QTL regions for control of SWG (Fig. 1). The first region included six DEGs and comprises the marker BT105286, which corresponds to the Norway spruce candidate gene MA_10428976g0010 (*PLAC8*), which was moderately upregulated at three dpi at the inoculation site.

The second QTL region on LG6 included 10 DEGs, all categorized as CCGs (Supplementary file 4). The previously validated marker *PaLAR3* (MA_176417g0010/ BT109050) is positioned in this QTL in the original linkage map^14^. However, the homologous marker (sp_v3.0_unigene4600) is positioned nearly 30 cM away from BT116508 in the composite map^25^. *PaLAR3* was not expressed in this study. However, the candidate gene MA_10433955g0020, encoding the homolog of the original QTL marker BT116508 also associated with SWG in the original study, is highly but not differentially expressed in this study (Table 2, Supplementary file 4). The candidate gene MA_853405g0010 (*hypothetical protein*) upregulated proximal to the inoculation at seven dpi (and three dpi) in response to inoculation, but it was not expressed in the distal samples collected from the inoculation treatment at seven dpi (Fig. 1). MA_122748g0010 (a putative *GRX, Glutaredoxin*) also showed significant up-regulation proximal to the inoculation in our RNAseq analysis. None of the candidate genes associated with the SWG QTL were upregulated distal to the inoculation site (Fig. 1).

**Table 2:** Identification and annotation of expressed candidate genes associated with the resistance to *H. parviporum* QTL regions in Norway spruce.

QTL			Identification of Norway spruce candidate genes			Annotation information		
LG	Position	Trait	Unigenes	Candidate genes	BLASTN	Annotation (Blast X)	E-value	sim mean (%)
1	151.7	IP	sp_v3.0_unigene1013	MA_10433492g0010	0	alanine aminotransferase 2	0	87.6
1	151.7	IP	sp_v3.0_unigene5249	MA_74668g0010	4E-144	uncharacterized protein LOC18442845-like	8.83715E-62	71.9
1	151.7	IP	sp_v3.0_unigene7019	MA_8609304g0010	0	RING-H2 finger ATL3	2.514E-139	62.9
1	151.7	IP	sp_v3.0_unigene9778	MA_3162g0020	0.001	probable E3 ubiquitin- ligase RNF217	4.1795E-115	62.3
1	151.7	IP	sp_v3.0_unigene2934	MA_138177g0010	1E-136	probable rRNA-processing EBP2 homolog	2.42956E-79	71.5
1	151.7	IP	sp_v3.0_unigene21078	MA_110169g0010	0	AAB01572.1 heat shock-like protein [Picea glauca]	7.71175E-41	81.9
1	161.3	E	sp_v3.0_unigene380	MA_106437g0010	0	Protein of unknown function (DUF789)	0	79.6
1	161.3	E	sp_v3.0_unigene4974	MA_125631g0010	0	Kinesin motor domain	0	91.4
1	162.1	E	sp_v3.0_unigene36567	MA_44157g0010	0	DNAJ heat shock family protein	1.78E-135	98.5
1	162.6	E	sp_v3.0_unigene11985	MA_585559g0010	0	Respiratory-chain NADH dehydrogenase 24 Kd subunit	3.21E-94	68.5
1	162.6	E	sp_v3.0_unigene16970	MA_90134g0010	7.30E-131	60S acidic ribosomal protein family	2.25E-32	60.5
2	75.6	IP	sp_v3.0_unigene109795	MA_186971g0010	5E-80	UDP-glycosyltransferase family protein	0	93.8
2	75.6	IP	sp_v3.0_unigene128417	MA_138834g0010	2E-144	aldehyde dehydrogenase family member	0	94.4
2	75.6	IP	sp_v3.0_unigene18333	MA_9478104g0010	0	rmlC-like cupin domain-containing	5.42113E-96	99.4
2	75.6	IP	sp_v3.0_unigene35076	MA_10434009g0010	0	AHW42454.1 GA2ox4, partial [Pinus tabuliformis]	0	89.6
2	81.7	E	sp_v3.0_unigene1167	MA_114154g0010	1.10E-164	Protein of unknown function (DUF1118)	2.98E-63	80.1
2	81.7	E	sp_v3.0_unigene17595	MA_10437148g0010	0	catalase 2	0	98.6
2	81.7	E	sp_v3.0_unigene30559	MA_10427042g0010	0	RING-variant domain	9.51E-05	78.1
2	82.7	E	sp_v3.0_unigene11933	MA_28209g0010	0	NAD(P)-binding Rossmann-fold superfamily protein	0	97.6
2	83.3	E	sp_v3.0_unigene182897	MA_10428567g0010	1.40E-145	Protein of unknown function (DUF679)	2.04E-113	92.5
2	84.6	E	sp_v3.0_unigene10696	MA_6091g0010	6.40E-90	–-NA–-	6.63E-40	89.9
2	85.5	E	sp_v3.0_unigene8898	MA_10435680g0010	0	Esterase/lipase/thioesterase family protein	6.83E-169	94.8
2	85.6	E	sp_v3.0_unigene126081	MA_120550g0010	0	Leucine-rich receptor-like protein kinase family protein	1.10E-138	85.5
2	86.2	E	sp_v3.0_unigene11941	MA_215978g0010	0	Eukaryotic aspartyl protease	0	89.9
2	86.2	E	sp_v3.0_unigene353	MA_140175g0010	0	Protein kinase superfamily protein	0	91.4
2	87.3	E	sp_v3.0_unigene126858	MA_218996g0010	3.70E-128	Oxygen evolving enhancer protein 3 (PsbQ)	4.38E-65	75.9
2	87.3	E	sp_v3.0_unigene17509	MA_10436080g0010	0	dehydroquinate dehydratase, putative / shikimate dehydrogenase, putative	1.81E-07	91.8
2	87.3	E	sp_v3.0_unigene17565	MA_10426048g0020	1.70E-80	alpha-galactosidase 2	1.63E-94	85.7
2	87.3	E	sp_v3.0_unigene7904	MA_10426424g0010	2.30E-134	Isocitrate dehydrogenase kinase/phosphatase (AceK)	1.34E-05	78.6
2	89.2	E	sp_v3.0_unigene27550	MA_48816g0010	0	universal stress PHOS32	1.79E-60	65.1
2	90.6	E	sp_v3.0_unigene30396	MA_10431443g0010	0	superoxide dismutase	1.69E-61	74.5
2	130.8	SWG	sp_v3.0_unigene14476	MA_129592g0010	0	receptor-like protein kinase 2	1.79E-06	57.7
2	131.8	SWG	sp_v3.0_unigene22490	MA_19032g0010	6.86E-102	unknown	3.03E-129	90.4
2	133.1	SWG	sp_v3.0_unigene8841	MA_27441g0010	4E-56	PREDICTED: glycerophosphodiester phosphodiesterase GDPD1, chloroplastic-like [Elaeis guineensis]	6.62E-17	62.1
2	134.4	SWG	sp_v3.0_unigene127279	MA_10427811g0010	1.3E-178	SRG1-like	1.7743E-150	75.9
3	75	E	sp_v3.0_unigene97367	MA_72133g0010	8.80E-82	CCAAT-binding transcription factor (CBF-B/NF-YA) subunit B	8.73E-28	98.3
3	75.7	E	sp_v3.0_unigene27952	MA_1548g0010	0	calmodulin-binding family protein	0	85.0
3	76.4	E	sp_v3.0_unigene34116	MA_15852g0010	0	phenylalanine ammonia-lyase 2	2.70E-64	79.4
6	32.9	SWG	sp_v3.0_unigene131072	MA_10434621g0010	4.00E-103	Ribosomal protein L32e	7.95E-50	91.4
6	41.1	SWG	sp_v3.0_unigene1862	MA_10428976g0010	0	PLAC8 family protein	0	67.7
6	42.1	SWG	sp_v3.0_unigene126822	MA_417026g0010	3.90E-118	Isochorismatase family	1.49E-82	86
6	42.1	SWG	sp_v3.0_unigene6079	MA_10430766g0010	0	Apolipophorin-III precursor (apoLp-III)	1.34E-87	85.5
6	42.9	SWG	sp_v3.0_unigene210326	MA_4442g0010	0	acyl-CoA oxidase 3	0	91.2
6	44.9	SWG	sp_v3.0_unigene209193	MA_12029g0010	0	Ribosomal protein L18ae/LX family protein	2.12E-125	98.3
6	111.6	E	sp_v3.0_unigene18249	MA_62177g0010	0	Phosphofructokinase family protein	3.83E-145	90.9
6	111.6	E	sp_v3.0_unigene34489	MA_10429833g0010	0	Protein of unknown function (DUF3529)	2.34E-172	94.4
6	112	E	sp_v3.0_unigene23234	MA_10264676g0010	5.00E-64	–-NA–-	1.27E-42	85.9
6	112.9	E	sp_v3.0_unigene2749	MA_213048g0010	2.20E-153	Histone superfamily protein	2.14E-31	65.6
6	113.5	E	sp_v3.0_unigene23240	MA_14341g0010	8.60E-113	Protein of unknown function (DUF581)	8.57E-66	67.6
6	115	E	sp_v3.0_unigene126925	MA_5176g0010	0	Myo-inositol-1-phosphate synthase	0.00E + 00	94.2
6	115	E	sp_v3.0_unigene30267	MA_101663g0010	5.50E-19	Oligosaccaryltransferase	1.45E-19	100
6	115.5	E	sp_v3.0_unigene16221	MA_10436501g0010	1.80E-147	unknown	5.71E-63	98.1
6	115.5	E	sp_v3.0_unigene27539	MA_10433186g0010	0	Ribonuclease T2 family	1.58E-155	93.3
6	117.1	E	sp_v3.0_unigene8468	MA_14707g0010	1.18E-08	ATP-dependent zinc metalloprotease FTSH chloroplastic	0	88.9
6	117.3	E	sp_v3.0_unigene3531	MA_25056g0010	0	NAP1-like protein	3.9317E-113	100.0
6	117.9	E	sp_v3.0_unigene22292	MA_264971g0010	5E-163	ATAF1-like protein, partial [Picea mariana]	3.042E-172	97.2
6	118.4	E	sp_v3.0_unigene6238	MA_1881g0010	0	acyl-coenzyme A oxidase peroxisomal	0	82.0
6	119.4	E	sp_v3.0_unigene6836	MA_181986g0010	0	Myb-like DNA-binding domain	0.00E + 00	96
6	119.7	E	sp_v3.0_unigene20354	MA_103386g0010	4E-92	ATAF1-like protein, partial [Picea mariana]	6.89E-136	85.4
6	119.7	E	sp_v3.0_unigene27482	MA_7719740g0010	2.05E-134	ABK24195.1 unknown [Picea sitchensis]	0	99.6
6	121.1	E	sp_v3.0_unigene22913	MA_16728g0010	0	transcription factor Trihelix family	2.19134E-89	63.0
6	125.7	E	sp_v3.0_unigene15800	MA_942991g0010	7E-13	EFTu, similar to Arabidopsis thaliana At4g20360	0	86.7
6	125.7	E	sp_v3.0_unigene36222	MA_10430642g0020	0.001	beta-fructofuranosidase like	3.52075E-63	72.7
6	125.9	E	sp_v3.0_unigene96752	MA_86256g0010	2.24E-62	ATAF1-like protein, partial [Picea mariana]	3.54E-115	79.9
6	125.9	E	sp_v3.0_unigene11986	MA_199901g0010	8E-131	unknown, similar to similar to Arabidopsis thaliana At1g18720	7.46E-133	98.5
6	126.6	E	sp_v3.0_unigene30362	MA_138328g0010	2.40E-88	–-NA–-	2.28E-35	90.1
6	126.6	E	sp_v3.0_unigene9511	MA_140162g0010	0	–-NA–-	6.65E-119	83.6
6	127.2	E	sp_v3.0_unigene10286	MA_96911g0010	0	Core histone	4.47E-119	82.7
6	127.3	E	sp_v3.0_unigene10376	MA_3262g0010	0	5′-AMP-activated protein kinase beta-2 subunit protein	5.16E-152	96.4
6	130.8	E	sp_v3.0_unigene23051	MA_98205g0010	0	F-box family protein	0.00E + 00	87.6
6	130.8	E	sp_v3.0_unigene36722	MA_120172g0010	4.60E-56	Splicing factor 3B subunit 10 (SF3b10)	1.25E-24	100
6	131.1	E	sp_v3.0_unigene4021	MA_137415g0010	4.90E-96	NAC domain containing protein 25	1.28E-31	78.7
6	131.9	E	sp_v3.0_unigene29556	MA_219331g0010	0	unknown	0.00E + 00	96.2
6	132	E	sp_v3.0_unigene126785	MA_10426681g0010	1.58E-85	Major intrinsic protein	2.19E-32	88.6
6	132	E	sp_v3.0_unigene29917	MA_268790g0010	1.25E-07	–-NA–-	1.27E-75	85.9
6	132	E	sp_v3.0_unigene18689	MA_9307243g0010	0	acyl-transferase family protein	0.00E + 00	95.8
6	132	E	sp_v3.0_unigene37742	MA_915221g0010	0	Pollen allergen	1.53E-138	88.6
6	134.4	E	sp_v3.0_unigene18634	MA_9055473g0010	0	Haloacid dehalogenase-like hydrolase (HAD) superfamily protein	0.00E + 00	69.3
6	135.8	E	sp_v3.0_unigene17528	MA_15246g0010	0	Zinc finger, C3HC4 type (RING finger)	2.77E-116	87.8
6	141.6	E	sp_v3.0_unigene10892	MA_10429538g0010	0	Polyketide cyclase / dehydrase and lipid transport	4.46E-123	97.3
6	141.6	E	sp_v3.0_unigene18104	MA_86745g0010	0	Thioredoxin	5.41E-109	98.2
6	141.6	E	sp_v3.0_unigene202138	MA_5692971g0010	4.60E-106	–-NA–-	5.56E-53	95.5
6	141.6	E	sp_v3.0_unigene34060	MA_10433807g0010	0	Multicopper oxidase	1.48E-108	81.1
6	141.6	E	sp_v3.0_unigene34081	MA_104063g0010	0	Zinc finger, C3HC4 type (RING finger)	2.34E-113	72.1
6	141.6	E	sp_v3.0_unigene36520	MA_134293g0010	3.00E-89	Cleavage site for pathogenic type III effector avirulence factor Avr	3.40E-40	77.1
6	142.2	E	sp_v3.0_unigene40516	MA_745622g0010	0	phosphoglycerate kinase	0.00E + 00	99.1
6	154	SWG	sp_v3.0_unigene18240	MA_10436145g0010	3E-41	F-box kelch-repeat At5g15710-like	0	49.8
6	154	SWG	sp_v3.0_unigene22702	MA_10434415g0010	1E-20	microtubule-associated RP EB family member	2.6854E-139	68.4
6	154	SWG	sp_v3.0_unigene32084	MA_10429904g0010	4E-85	ABK22325.1 unknown [Picea sitchensis]	1.56689E-71	83.0
6	154	SWG	sp_v3.0_unigene6593	MA_10230764g0010	8E-17	ABR17176.1|unknown [Picea sitchensis]	6.1607E-135	64.3
6	154	SWG	sp_v3.0_unigene73878	MA_131587g0010	0	chlorophyll a b-binding	8.8049E-161	98.4
6	154	SWG	sp_v3.0_unigene86259	MA_853405g0010	1.91E-32	hypothetical protein [Pinus taeda]	3.0779E-39	71.7
6	154	SWG	sp_v3.0_unigene22630	MA_248791g0010	2E-113	unknown [Picea sitchensis]ABK23762.1	1.5646E-149	100.0
6	154	SWG	sp_v3.0_unigene18288	MA_962052g0010	0	PREDICTED: haloacid dehalogenase-like hydrolase domain-containing protein 3 [Prunus mume]	1.67E-120	84.9
6	154	SWG	sp_v3.0_unigene13063	MA_10426138g0010	1.23E-117	dihydropyrimidinase isoform	1.1E-143	91.4
6	154	SWG	sp_v3.0_unigene5833	MA_122748g0010	8E-88	Glutaredoxin	0	99.2
8	140.8	LL	sp_v3.0_unigene21297	MA_184192g0010	0.00E + 00	tubulin alpha-2 chain	0.00E + 00	92.6
8	142.4	LL	sp_v3.0_unigene12232	MA_52380g0010	0	UDP-glucuronate 4-epimerase 3	0	86.2
8	143	LL	sp_v3.0_unigene10846	MA_10435810g0010	0	cinnamoyl- reductase 2	7.9337E-140	79.8
8	143.4	LL	sp_v3.0_unigene13707	MA_107074g0010	3E-117	F-box At5g51380-like	1.2968E-180	77.8
8	143.4	LL	sp_v3.0_unigene17253	MA_10432221g0010	8E-50	adenine phosphoribosyltransferase like	6.47166E-51	94.2
8	143.4	LL	sp_v3.0_unigene26050	MA_10431633g0010	5E-14	ADE77033.1 unknown [Picea sitchensis]	7.0318E-142	71.5
8	144.7	LL	sp_v3.0_unigene7190	MA_10296151g0010	0.00E + 00	Carboxylesterase family	0.00E + 00	92.9
8	145.3	LL	sp_v3.0_unigene61649	MA_17691g0010	3.20E-113	Leucine-rich repeat protein kinase family protein	9.95E-06	68.6
9	122.4	SWG	sp_v3.0_unigene3494	MA_10429563g0010	0	Protein kinase domain	6.62E-155	98.2
9	126.3	SWG	sp_v3.0_unigene38053	MA_3650g0010	0	CAB06080.1 porin [Picea abies]	0	94.9
9	126.3	SWG	sp_v3.0_unigene36841	MA_19215g0010	0	–-NA–-	–-NA–-	–-NA–-
9	127.2	SWG	sp_v3.0_unigene30750	MA_108756g0010	8.35E-65	NRT3 family	1.4193E-122	70.2
9	127.5	SWG	sp_v3.0_unigene182972	MA_10435879g0010	4.62E-145	NAD(P)-binding Rossmann-fold superfamily	0	92.9
11	30	IP	sp_v3.0_unigene18400	MA_362678g0010	0	S-adenosyl-L-methionine-dependent methyltransferases superfamily protein	2.20E-164	99.2
11	30.1	IP	sp_v3.0_unigene30128	MA_10018366g0010	5.95E-76	–-NA–-	3.84E-30	100.0
11	31.2	IP	sp_v3.0_unigene207722	MA_427046g0010	1.00E-83	Core histone H2A/H2B/H3/H4	1.56E-36	95.7
11	31.7	IP	sp_v3.0_unigene8310	MA_215979g0010	1E-102	ABK21694.1 unknown [Picea sitchensis]	3.8437E-123	98.8
11	32.3	IP	sp_v3.0_unigene162285	MA_24271g0020	1E-167	R2R3-MYB transcription factor PgMYB11-like	0	96.9
11	32.3	IP	sp_v3.0_unigene15787	MA_6931g0010	0.0000586	caffeoyl CoA O-methyltransferase [Picea abies]	1.9055E-169	99.2
11	32.4	IP	sp_v3.0_unigene18329	MA_4742g0010	9E-111	MFT1-like protein [Picea abies]	1.783E-125	99.4
11	32.5	IP	sp_v3.0_unigene4041	MA_7247276g0010	1E-152	cytochrome P450 monooxygenase CYP736B	0	95.5
11	32.5	IP	sp_v3.0_unigene4998	MA_10427673g0020	0	cytochrome P450 CYP736A12-like	3.03E-75	73.6
11	33.3	IP	sp_v3.0_unigene9613	MA_14663g0020	0	Cytochrome P450-like,	0	98.4
11	36.4	IP	sp_v3.0_unigene25068	MA_498632g0010	0	2-oxoglutarate (2OG) and Fe(II)-dependent oxygenase superfamily protein	5.85E-180	86.3
11	36.4	IP	sp_v3.0_unigene53315	MA_10425962g0020	2.20E-99	2-oxoglutarate (2OG) and Fe(II)-dependent oxygenase superfamily protein	2.69E-91	90.1
11	36.8	IP	sp_v3.0_unigene9722	MA_28222g0010	1.80E-180	Cytochrome P450 superfamily protein	0	86.1
11	40.1	IP	sp_v3.0_unigene36253	MA_19954g0010	0	unknown	1.53E-17	83.2
11	40.1	IP	sp_v3.0_unigene9396	MA_139843g0010	0	Tetraspanin family	4.37E-07	79.2

^a^ Linkage groups are numbered according to Lind et al.^14^; ^b^ Position of QTL regions for traits controlling resistance to *H. parviporum*;^c^ QTL regions for traits controlling resistance to *H. parviporum* as described in Lind et al.^14^: exclusion (E), infection prevention (IP), lesion length (LL), sapwood growth of fungus (SWG); ^d^ unigenes according to SustainPine v3.0;^e^ unique candidate genes in Norway spruce genome *Pabies* v1.0; ^f^blastN score of the best hit in Norway spruce genome *Pabies* v1.0; ^g^ Best BlastX hit of the Norway spruce candidate genes in Genbank nr protein database.

On LG9, we found 15 expressed candidate genes in the SWG QTL region (Supplementary file 4), five of these were also differentially expressed, including MA_19215g0010, the homolog of the original marker BT115393. All the DEGs in this region showed higher expression after wounding than after inoculation with *H. parviporum* (Fig. 1).

### DEGs in QTLs associated with lesion length

The only original marker (02739-B22-309) we could identify with the QTL for lesion length in the phloem included in the *Pinaceae* composite map was located on LG8. We found 13 candidate genes that were expressed in this region, and eight of these were differentially expressed. All of the candidate genes except *UDP-glucuronate 4-epimerase* (MA_52380g0010), which was induced at seven dpi at site A and C respectively, were downregulated in response to inoculation (Fig. 1, Supplementary file 4). A *Leucine-rich repeat protein kinase family protein* gene (MA_17691g0010) was down regulated proximal to inoculation site at three and seven dpi. *UDP-glucuronate 4-epimerase* and *Leucine-rich repeat protein kinase family protein* gene were categorized as PCGs. It is noteworthy that a *cinnamoyl-CoA reductase* (MA_10435810g0010) was repressed at seven dpi at both proximal and distal to the inoculation. It is also the only candidate gene in CCG category (Fig. 1, Supplementary file 4).

### DEGs in QTLs associated with exclusion of the *H. parviporum* from the Norway spruce

We could identify the QTLs associated with exclusion of the fungus from the host located on LG1, LG2, LG3 and LG6^14^ in the composite map. On LG1 five DEGs were found and two DEGs of which were upregulated at seven dpi one at proximal and other at distal site (Fig. 1, Supplementary file 4).

On LG2 we found 16 DEGs associated with the QTL and only one of these is a CCG, which is the homolog of the original QTL marker (BT100742) MA_10431443g0010 a *superoxide dismutase* (Fig. 1, Table 2, Supplementary file 4). Both MA_10431443g0010 and MA_48816g0010 were downregulated proximal to the inoculation site at seven dpi. A *Catalase* (MA_10437148g0010) and *Esterase family protein* (MA_10435680g0010) were induced at seven dpi at proximal site and at distal site respectively (Fig. 1, Supplementary file 4). The candidate gene MA_28209g0010 (*NAD(P)-binding Rossmann-fold superfamily protein*) was upregulated proximal and distal to the inoculation site at both three and seven dpi. It was notable that candidate gene (MA_10436080g0010) *dehydroquinate dehydratase, / shikimate dehydrogenase* was induced in response to inoculation in all conditions tested (Fig. 1, Supplementary file 4).

There were three DEGs associated with the exclusion QTL on LG3. Interestingly, *phenylalanine ammonia-lyase* (*PAL*) (MA_15852g0010), an ortholog of the *Picea glauca PgPAL3* (Genbank: BT119163)^35^ identified as PCG, was upregulated at both proximal and distal site; while the other two were downregulated proximal to inoculation, at both three and seven dpi (Fig. 1, Supplementary file 4).

**Figure 2:**
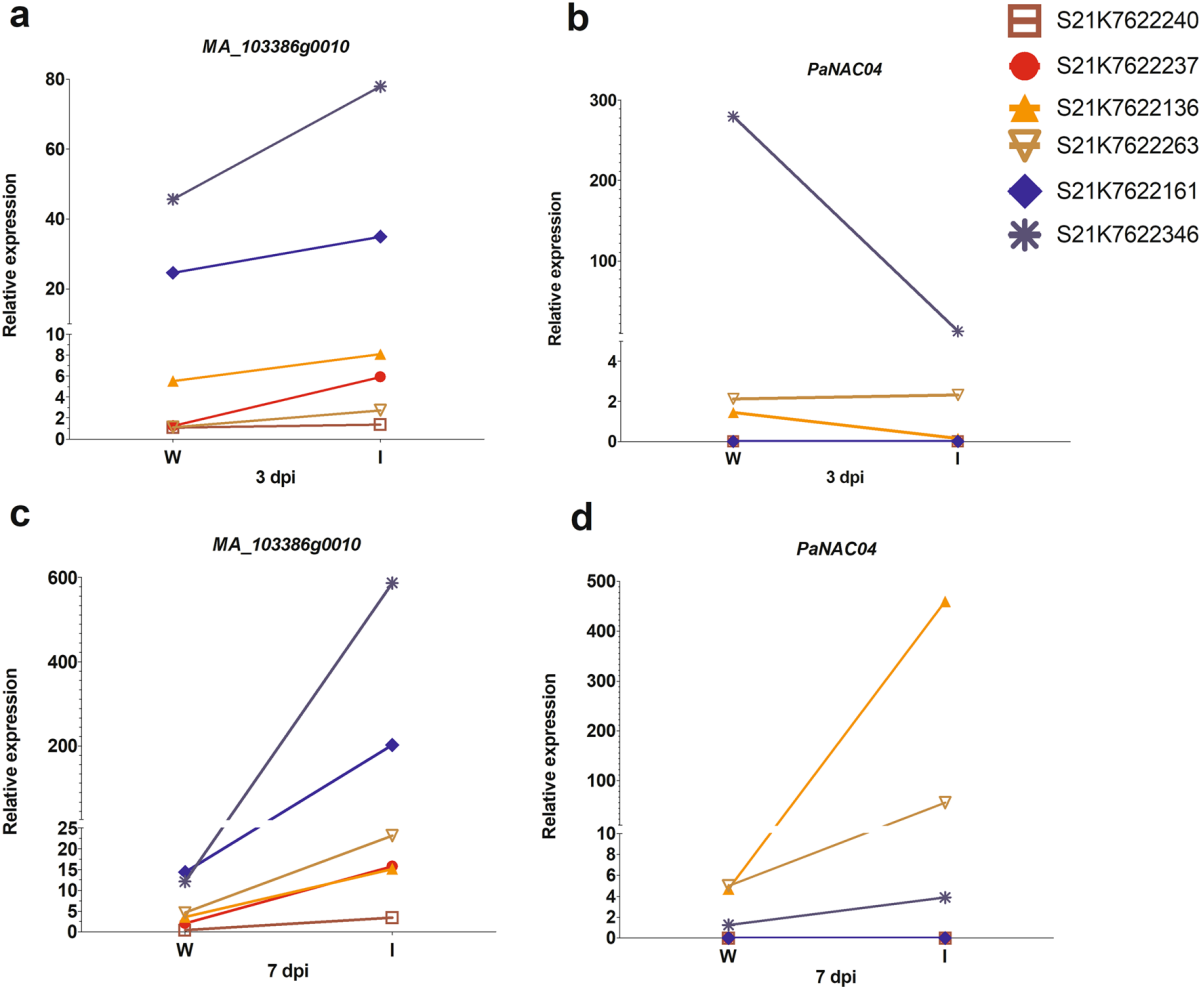
qRT-PCR of NAC candidate gene (MA_103386g0010 and *PaNAC04*) in bark of 7 year old genotypes of Norway spruce in response to wounded and inoculated with *H. parviporum*. The data have been normalized to the constitutive expressed genes *Elongation-factor-1*α and *Elongation-factor-4* α*.* Different symbols with different colours represent six well characterized Norway spruce genotypes. (**a**) and (**c**) shows relative expression pattern of MA_103386g0010 at 3 and 7 dpi and (**b**) and (**d**) shows relative expression pattern of *PaNAC04* at 3 and 7 dpi, in response to wounding and inoculation (N = 6).

Like with the second QTL for SWG on LG6, the exclusion QTL on LG6 had a large number of expressed CCGs (19) and 72 PCGs. Forty two of these candidate genes were also differentially expressed in at least one comparison (Fig. 1, Supplementary file 4). The orthologs (MA_14707g0010, MA_16728g0010 and MA_942991g0010) of the three markers associated with the original QTL, all showed differential expression in this study (Fig. 1). Interestingly enough, the QTL region for exclusion on LG6 showed three candidate genes encoding class III-3 NAC transcription factors: MA_264971g0010 (*PaNAC04*), MA_86256g0010 and MA_103386g0010. The candidate gene *PaNAC04* and MA_103386g0010 were classified as CCGs. However, MA_86256g0010 was classified as a PCG. All three candidate genes were relatively highly expressed and showed clear induction, both proximal and distal, to the inoculation compared to wounding. This was especially noticeable at one week after the inoculation (Fig. 1), although, only *PaNAC04* was differentially regulated in S21K0220184 distally from the inoculation site at seven dpi (Fig. 1, Supplementary file 4). This expression pattern together with the previously published phylogeny of the sub group III-3 NACs^29^ led us to investigate if the MA_103386g0010 candidate gene represents a different gene from the previously described *PaNAC04.* Additionally, an analysis of *PaNAC04* (MA_264971g0010) and MA_103386g0010 expression by qRT-PCR in six well characterized Norway spruce genotypes^23^ showed that, on average *PaNAC04* was not significantly differentially expressed. However, MA_103386g0010 was differentially expressed at seven dpi in response to inoculation (Table 3). Furthermore, expression of *PaNAC04* was only detected in three of the genotypes (Fig. 2b, d) whereas MA_103386g0010 was expressed in all genotypes and treatments (Fig. 2a, c).

**Table 3:** Comparison of log_2_ fold change of the genes in RNAseq and qRT-PCR.

Genes	RNA-seq log_2_ (fold change)	RNA-seq log_2_ (fold change)	qPCR log_2_ (fold change)	qPCR log_2_ (fold change)	Annotation
	I3 vs W3	I7 vs W7	I3 vs W3	I7 vs W7	
MA_103386g0010	1.61*	2.81*	0.68	4.51*	NAC- transcription factor
MA_264971g0010	1.93*	2.75*	0.80	5.58	NAC- transcription factor

Comparison of expression patterns of candidate genes in RNAseq and qRT-PCR experiment. The value represent log_2_ fold change in inoculation compared to wounding at 3 and 7 days post inoculation. I3 and W3 means inoculation and wounding at 3 dpi and I7 and W7 means inoculation and wounding at 7 dpi. Asterisks (*) indicate significant higher induction level in *H. parviporum* compared to wounding alone (P < 0.05).

## Discussion

Throughout evolution, species in the *Pinaceae* have maintained a high degree of synteny and collinearity in their genomes^25,26,36–38^. In this study we have capitalized on this feature to expand the array of potential candidate genes associated with the resistance QTLs originally reported by Lind and co-workers^14^. As the composite map^25^ is considerably more dense than the original map, it may allow for identification of additional candidate genes associated with the reported QTL regions. Using shared markers associated with the QTL regions, identified around a significant LOD peak inside the confidence interval and subsequent markers outside the LOD peak in the *Pinaceae* composite map^25^, we found 329 potential Norway spruce candidates associated with 12 out of 13 original QTLs. Two hundred and ninety eight of the candidate genes were expressed, of which 80 were within confidence interval candidate genes (CCGs) and 218 putative candidate genes (PCGs). Out of these 41 CCGs and 83 PCGs were differentially expressed in the RNAseq study.

The products of the candidate genes could affect the resistance trait either as part of the constitutive or the induced defence^39,40^. Candidate genes involved in constitutive defence response are important for the host, but were not included in our current study. Compared to induced candidate genes associated with induced defences, whose involvement can be detected by transcriptional, protein or metabolite accumulation the contribution of constitutive defences are very difficult to quantify in short-term experiments. Therefore, we delibrately chose to focus on identifying candidate genes with a potential role in the induced defences in Norway spruce. It is reasonable to hypothesize that candidate genes associated with the resistance QTLs and also showing differential regulation in response to *H. parviporum* inoculation may be connected to the resistance phenotype^15,24^. In this study we used progenies from one Norway spruce cross and one *H. parviporum* strain to create expression profiles of the candidate genes associated with the resistance QTLs. Plant materials used in this study was represented by a set of closely-related individuals drawn from the original QTL population^11,14^. This approach may have limitations as the use of more host and pathogen genotypes could have given a broader picture*.* However, in this study we wanted to study the expression pattern of the candidate genes mapped in the original QTL mapping study^14^ in response to same fungal isolate *H. parviporum* Rb175 that was used to detect the QTLs. Using more *H. parviporum* isolates in the study would have perhaps generated information on how broad the transcriptional responses of the candidate genes associated with the QTLs were. Even though the choice to use full-sib plants and *H. parviporum* Rb175 might have the drawback of not being able to identify some of the broader defence responses in Norway spruce, it can help removing “signal noise” as resistance against *H. parviporum* in Norway spruce is a quantitative trait with multiple genes having small effect where highly resistant individuals might rely on a different set of genetic factors. The approach in this study allowed us to work with well-studied plant materials and to use a narrow genetic base to generate initial data^41,42^ and identify potential resistance candidates for further work, which was one of the primary objectives of this study. Clearly we missed out on potential candidate genes which are not associated with the QTL regions in the genetic linkage map and also candidates associated with QTLs which are not present in the original study^43,44^. Nevertheless, combining genomic and transcriptomic analysis we identified 124 candidate resistance genes which could be considered as candidates for induced resistance.

Fungal sapwood growth (SWG) is a trait that reflects the trees capacity to restrict the spread of the pathogen in its sapwood. The general assumption is that the trees with shorter SWG after inoculation with *H. annosum* s.l. in the sapwood would also display shorter decay columns after natural infections^45^. We could locate all of the original QTLs for SWG in the composite map^14,25^. Interestingly, the inspection of the QTL for SWG2 on LG6 including the previously validated marker *PaLAR3*^15^ in the composite map, positioned *PaLAR3* nearly 30 cM away from the other markers in this QTL region. This could suggest that the markers found around 154 cM on LG6, including BT116508/ MA_10433955g0020 encoding magnesium-protoporphyrin, may not be linked to the original SWG QTL or the original QTL^14^ comprises several independent QTLs which could not be separated due to low density of markers in this QTL region. Despite this we chose to include these candidate genes in the subsequent analyses, and 39 expressed candidate genes associated with the SWG QTL were identified. In fact, 16 of the candidate genes differentially expressed (10 CCGs and 6 PCGs) in response to inoculation were found at this position in the map. Among the upregulated candidate genes associated with the SWG QTLs MA_853405g0010 (a hypothetical protein containing a domain of unknown function, DUF4228), and the MA_122748g0010 (a putative *GRX, Glutaredoxin*) showed significant up-regulation during inoculation compared to wounding at both three and seven dpi in our RNAseq analysis. Both of these DEGs were positioned at 154 cM on LG6. The expression patterns of the candidate genes in this region could possibly indicate that they are involved in the expression of the resistance trait. Clearly, fine mapping the region between BT116508 and BT109050/*PaLAR3* would be very helpful to resolve the locus structure.

One of the two QTLs for lesion length (LL) could be identified in the composite map^14,25^. We found two DEGs associated with cell wall modifications and specialized metabolism in this QTL region. The moderately upregulated DEG MA_52380g0010 identified as PCG (log_2_ Fold Change of 0.75) with similarity to *UDP-glucuronate 4-epimerase* that presumably catalyzes the formation of the key building block of pectins, UDP-d-galacturonic acid^46,47^. The second identified as CCG in this QTL that is putatively associated with cell wall modifications encodes a CCR-like protein (cinnamoyl-CoA reductase, MA_10435810g0010) and was repressed both proximally and distally at seven dpi. CCR is the first committed enzyme in the lignin-specific pathway^48,49^, and it is possible that the downregulation of this gene is part of a redirection of resources away from the lignin biosynthesis pathway to other branches of the phenolics metabolism (see also discussion on NAC transcription factors below). The repressed *CCR* gene would thus be in line with Norway spruce allocating more resources to potential antifungal low molecular weight phenolics following challenges with *H. annosum*^24^. It has been shown that suppression of *CCR* gene expression in Norway spruce decreases lignin content^50^. Suppression of *CCR* gene expression in tobacco has been accompanied by accumulation of phenolic substances^51,52^. Further analyses, eg. with RNAi- or overexpression constructs would shed light on cinnamoyl-CoA reductase’s (MA_10435810g0010) role in the interaction with *H. parviporum*.

There are two expression hotspots associated with the QTL regions for IP and E. The traits IP and E are measures of the ability the host have to stop the fungus from entering the wound upon inoculation or the ability to hem in and exclude an invading pathogen^14^. Thus, these traits could potentially reflect the “true” resistance to infection, and consequently the candidate genes associated with these QTLs were of special interest to us. Two thirds of the expressed genes as well as differentially expressed genes were associated with these QTLs. It is difficult to decide when to best capture gene expression patterns associated with IP and E in the artificial inoculation system we employ, as the read out of the traits takes place after several weeks of interaction^14,53^. Consequently, the expression data gathered for these traits should be seen as a probe and genes not differentially expressed in the current study could still be highly relevant to induced defence responses in *H. parviporum* resistance.

It is notable that several of the candidate genes associating with the QTL regions for IP are genes or orthologs to genes, which have been shown to respond to biotic stress^29,54^ or control other adaptive traits^55,56^ in the genus *Picea.* MA_24271g0020 (*PgMYB11*-like), which corresponds to the original marker BT103501 in the first QTL for IP on LG11^14^, is one example. This marker has been found to associate with a QTL for bud set in black spruce^55^. The observation that variation in BT103501 associate with two apparently different traits in spruce, indicates pleiotropic effect or tightly linked loci^55,57^. Another of the original markers in this QTL MA_4742g0010 (PabiesFT1-1,251/BT115191) encoding a spruce Mother of FT1- like protein MFT1, suggested to control the formation of resin ducts in male buds^56^. MA_4742g0010 was weakly expressed but significantly upregulated at seven dpi both proximal and distal to the inoculation. Neither *PgMYB11*-like nor Norway spruce *MFT1*, have been connected to host defence previously. In contrast, a DEG associated with this QTL that has a recognized role in the induced defences in conifers is MA_6931g0010. This candidate gene encodes a putative *caffeoyl-CoA O-methyltransferase* (*CCoAOMT*) and it is highly expressed in all treatments and was weakly upregulated at seven dpi proximal to the inoculation site. *CCoAOMT1,* an enzyme in the lignin biosynthesis pathway^54^ has been implicated in budworm and white pine weevil resistance in white spruce.

The candidate gene *Phenylalanine ammonia-lyase* (*PaPAL3*) identified as a PCG (MA_15852g0010), an ortholog of the *Picea glauca PgPAL3* (Genbank: BT119163)^35^ at the QTL for exclusion on the LG3, was upregulated both proximal and distal to the inoculation site at three and seven dpi. Two other *PaPAL1* and *PaPAL2* is reported to be induced upon wounding and *H. annosum s.l.*^24,58,59^. PAL is the first enzyme committed in the phenylpropanoid biosynthesis pathway^60^. The activation of phenylpropanoid biosynthetic pathway which leads to the production of polyphenolics. Flavonoids and stilbene monomers plays a central role in the induced defence towards wounding and fungal infection in conifers^61–64^. Stilbene astringin was negatively correlated with the depth of the hyphal penetration of *Heterobasidion annosum* in Norway spruce bark^65^. Flavonoids have an antimicrobial effect on *H. annosum* s.l.^24^ and *E. polonica*^20,61–63^ in Norway spruce.

Just like the IP QTL on LG11, the QTL region for fungal exclusion from the sapwood on LG6 also involved a number of candidate genes that had been studied previously in conifers. However, the most interesting feature of the QTL region for exclusion on LG6 is that it harbours three of the previously identified seven Norway spruce candidate genes with similarity to subgroup III-3 NAC transcription factors^29^; *PaNAC04*, MA_86256g0010 and MA_103386g0010. All three candidate genes were relatively highly expressed and showed clear upregulation in response to inoculation with *H. parviporum* both proximal and distal to the inoculation (Fig. 1). Their upregulation could possibly be associated with a shifted balance from cell wall use to defense active phenylpropanoids^29^. Naturally, the question arose if these three predicted candidate genes indeed represent distinct genes. The difficulties in assembling the large and repetitive conifer genomes into scaffolds will lead to errors in the assembly^66,67^. Thus, it would not be unlikely that the three highly similar candidate genes^29^, correspond to one single gene located in the Exclusion QTL on LG6. However, based on the expression patterns detected by qRT-PCR, which agrees with the previous phylogenetic analysis^29^, placing *PaNAC04* and MA_86256g0010 together on a supported branch separate from MA_103386p0010 in subgroup III-3 of the NAC transcription factor family, we argue that there are at least two NAC genes associated with this QTL. Albeit, tightly linked and not much diverged, but this must be confirmed by resequencing of the region.

This study gives an insight into Norway spruce genome organization with information of position of the candidate genes associated with resistant trait in the genome e.g. the previously described *PaNAC04. PaNAC04* was not only upregulated in response to *H. parviporum* but it was also located in the region important for controlling resistance determined by QTL mapping^14^. Therefore, allelic variation in *PaNAC04* needs to be further studied in future experiment to understand the role of *PaNAC04* in controlling induce defence response.

In conclusion; this study, combining map-based information and expression analyses have associated previously identified candidate genes, such as *PaNAC04* and MA_103386g0010, with genomic regions in Norway spruce harboring resistance QTL, strengthening their predicted role in control of *H. annosum s.l.* infection. This approach has also allowed us to identify a set of novel candidate genes for future analyses, the most prominent being genes associated with the phenylpropanoid pathway *CCR* (MA_10435810g0010), the *PgMYB11*-like gene and *PAL* (MA_15852g0010).

## Supplementary information

Supplementary file1

Supplementary file2

Supplementary file3

Supplementary file4

Supplementary file5
