# Supplementary material for: Combining transcriptomics and genetic linkage based information to identify candidate genes associated with Heterobasidion-resistance in Norway spruce
Source: Sci Rep. 2020 Jul 29;10:12711. doi: 10.1038/s41598-020-69386-0 (PMC7391732; doi:10.1038/s41598-020-69386-0)
Supplement: Supplementary file 5 — Supplementary file5 [file 41598_2020_69386_MOESM5_ESM.pdf]

Supplementary Information

Article in Scientific Reports

Combining transcriptomics and genetic linkage based information to identify candidate genes associated with *Heterobasidion*-resistance in Norway spruce

Rajiv Chaudhary[1*], Karl Lundén[1+], Kerstin Dalman[1,2+], Mukesh Dubey[1], Miguel Nemesio-Gorriz[1,3], Bo Karlsson[4], Jan Stenlid[1] and Malin Elfstrand[1]

[1]Department of Forest Mycology and Plant Pathology, Swedish University of Agricultural Sciences, Box 7026, 75007 Uppsala, Sweden; [2]Department of Molecular Sciences, Swedish University of Agricultural Sciences, SE-75007, Uppsala, Sweden; [3]Teagasc Food Research Centre, Ashtown, Dublin 15, D15DY05, Ireland; [4]The Forestry Research Institute of Sweden, Ekebo, SE-268 90, Svalöv, Sweden

(* contact person: rajiv.chaudhary@slu.se)

Supplementary table 1: Primer sequence used for qPCR

**Genes**	**Forward Primer**	**Reverse primer**	**Annotation**
MA_103386g0010	ACCTTCTCCAAC AAGCCACAC	ACTGGAGCCTTG AGGGTAGTGG	NAC-Transcription factor
MA_264971g0010	GGACCTTCGACC ACAAGCTATAG	ACTGGAGTCTTAA GGGTACTGC	NAC-Transcription factor (*PaNAC04*)
elF4A	AGTAAGCCCGTG AGGATTC	AGTCAGCCAGTC AACCTTTC	Reference gene
ELF1α	TGGCAAGGAACT GGAGAAGGAA	TAGTCCCTCACAG CAAAACGA	Reference gene

Supplementary table 2: Differential expressed genes in inoculation or wounding at proximal and distal site.

Conditions	DEGs	Up-regulated in inoculation	Down-regulated in inoculation
3A_I vs 3A_W	6999	4562	2437
7A_I vs 7A_W	8767	4976	3791
7C_I vs 7C_W	4401	1723	2678

Supplementary table 3: Distribution of expressed and differential expressed candidate genes in QTL regions of Norway spruce associated with *H. parviporum* resistance

LG[a]	Trait[b]	CCG[c] Expressed genes	CCG[c] DEGs	PCG[d] Expressed genes	PCG[d] DEGs
LG1	IP	12	6	0	0
	E	3	2	13	3
LG2	IP	12	4	3	0
	E	3	1	36	15
	SWG	1	0	11	4
LG3	E	1	0	16	3
LG6	SWG1	1	1	22	5
	E	19	10	72	32
	SWG2	13	10	3	0
LG8	LL	1	1	12	7
LG9	SWG	8	3	7	2
LG11	IP1	5	3	23	12
	IP2	1	0	0	0
Total		80	41	218	83

[a] Linkage groups are numbered according to the Lind et al 2014; [b] QTL regions for traits controlling resistance to *H. parviporum* as described in Lind et al 2014: exclusion (E), infection prevention (IP), lesion length (LL), sapwood growth of fungus (SWG); [c] the number expressed and DEGs of confidence interval (CCG) in the QTL regions [d], the number of expressed and DEGs putative candidate genes (PCG) outside confidence interval by de Miguel et al 2015.
